# Long-Term Outcomes of the PRESERFLO MicroShunt Implant in a Heterogeneous Glaucoma Cohort

**DOI:** 10.3390/jcm12134474

**Published:** 2023-07-04

**Authors:** Jens Julian Storp, Friederike Elisabeth Vietmeier, Ralph-Laurent Merté, Raphael Koch, Julian Alexander Zimmermann, Nicole Eter, Viktoria Constanze Brücher

**Affiliations:** 1 Department of Ophthalmology, University of Muenster Medical Center, 48149 Muenster, Germany; friederikeelisabeth.vietmeier@ukmuenster.de (F.E.V.); ralph-laurent.merte@ukmuenster.de (R.-L.M.); julian.zimmermann@ukmuenster.de (J.A.Z.); nicole.eter@ukmuenster.de (N.E.); viktoria.bruecher@ukmuenster.de (V.C.B.); 2 Institute of Biostatistics and Clinical Research, University of Muenster, 48149 Muenster, Germany; raphael.koch@ukmuenster.de

**Keywords:** success, failure, MIGS, microinvasive glaucoma surgery, intraocular pressure, IOP, real-world, PEX, POAG, secondary

## Abstract

The Preserflo MicroShunt represents a novel glaucoma treatment device, necessitating long-term follow-up data to accurately assess its efficacy. The aim of this study is to report real-world data of a heterogenous glaucoma cohort who received Preserflo implantation at a specialized glaucoma clinic. A total of 160 eyes of 160 patients who underwent Preserflo MicroShunt implantation were retrospectively enrolled in this study. Patient characteristics, as well as success and failure rates, were assessed. The numbers of adverse events and revision procedures were recorded, along with any reduction in supplementary medication. The progression of intraocular pressure (IOP) was assessed over the course of 12 months, and fluctuations were analyzed. The overall success rate was 61.9% (complete success: 51.3%, qualified success: 10.6%). Revision surgery was performed in 25% of cases. Excessive hypotony occurred postoperatively in 54.4% of patients and regressed after 7 days in 88.8% of all cases. Median IOP decreased from 22 (interquartile range (IQR): 17–27) mmHg preoperatively to 14 (IQR 12–16) mmHg at 12 months postoperatively (*p* < 0.01). The median number of antiglaucomatous agents decreased from three to zero at latest follow-up. The Preserflo MicroShunt achieved a noticeable reduction in IOP over the course of 12 months in glaucoma patients, irrespective of disease severity or disease subtype. The frequency of postoperative adverse events and number for revision surgeries over the course of the follow-up period were low.

## 1. Introduction

Glaucoma is a worldwide leading cause of blindness [1]. An increased intraocular pressure (IOP) is regarded as one of the main risk factors associated with the disease [2], which is why the majority of treatment options focus on IOP reduction. The latter can be achieved via the administration of IOP lowering drugs, nonpenetrating approaches, such as laser procedures, or penetrating surgical intervention. To date, trabeculectomy remains the gold standard for penetrating glaucoma surgery [3,4,5], despite requiring intensive post-surgical follow-up [4,5]. Microinvasive glaucoma surgery (MIGS) treatment options are intended to provide an acceptable IOP reduction for patients, while reducing intra- and postoperative care burden [5].

Recent years have seen a number of these MIGS devices being developed. One novel innovation is the Preserflo MicroShunt (Santen, Miami, FL, USA), an 8.5 mm long tubular structure with a 350 µm outer diameter and 70 µm lumen made from biocompatible (poly)styrene-block-isobutylene-block-styrene [5,6]. The Preserflo system is placed into the subconjunctival space and acts as a drainage device, transporting aqueous humor from the anterior chamber to the subconjunctival space. The system has been reported to lower IOP during long-term observation in different types of glaucoma [7,8,9,10]. Due to its size, the Preserflo MicroShunt is expected to be associated with a faster recovery time and fewer postoperative adverse events in comparison to larger penetrating interventions, such as trabeculectomy [10]. 

Although a number of studies have already demonstrated the effectiveness of the Preserflo system [9,10,11], further studies are needed to assess long-term efficacy in clinical settings. This is especially true, as most of the studies available today investigated the efficacy of the Preserflo system in open-angle glaucoma patients only [8,12,13]. Yet, in clinical routine, practitioners might want to be able to offer any patient surgical therapy using the Preserflo system, regardless of the type of glaucoma. Therefore, studies reporting real-world data for heterogeneous glaucoma populations are required. In this study, we report real-world outcomes of the Preserflo MicroShunt in a large patient cohort consisting of patients with various types of glaucoma and disease severity levels. 

This study investigates success rates, failure rates, the decrease in intraocular pressure (IOP), and the clinical development observed in a glaucoma patient group who underwent Preserflo implantation at a specialized glaucoma clinic in Germany over a 1 year period.

## 2. Materials and Methods 

### 2.1. Design and Setting

All procedures were performed in accordance with the ethical standards issued by the ethics committee of the Medical Association of Westfalen-Lippe and the University of Münster, as well as the 1964 Helsinki declaration and its later amendments. Informed consent was waived due to the retrospective nature of this study. Data in this retrospective, monocentric trial were collected from glaucoma patients visiting the Department of Ophthalmology at the University Hospital Münster, Germany from July 2020 to December 2022. Data were obtained from electronic patient records in the digital documentation system FIDUS (Arztservice Wente GmbH, Darmstadt, Germany). 

All patients older than 18 years of age, who received implantation of the Preserflo MicroShunt during this time span, were eligible for study inclusion. In accordance with the guidelines of the World Glaucoma Association, all fellow eye surgeries were excluded from the database of this study [14].

### 2.2. Surgical Procedure 

**Figure 1:**
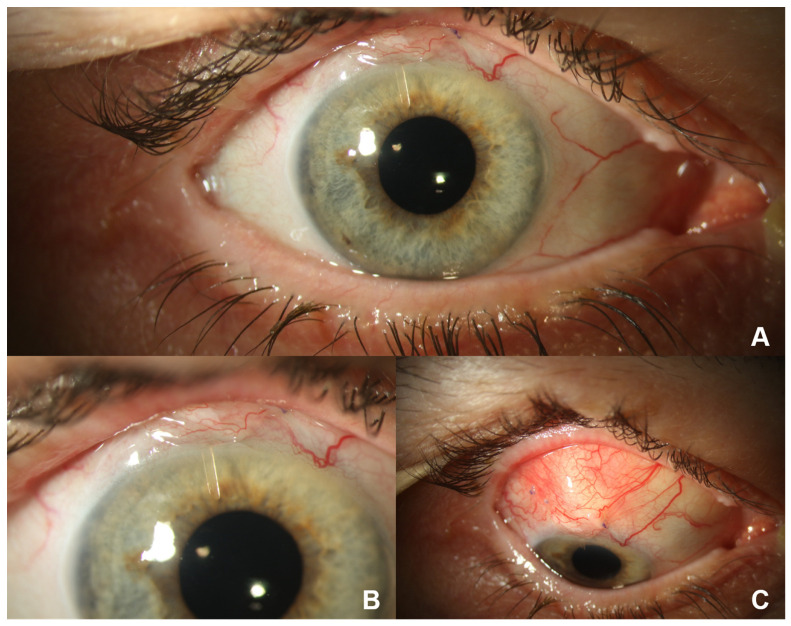
Situs after Preserflo Microshunt implantation in a right eye 1 day after surgery. (**A**) Macroscopic view: the scleral pocket is partly covered by the upper eyelid. (**B**) Close-up view: the shunt reaches into the anterior chamber without touching the iris or cornea. The scleral pocket is partly covered by the upper eyelid. (**C**) View of the prominent scleral pocket during downward gaze.

In the preoperative phase, patients at our clinic stop taking any antiglaucomatous eye drops 4 weeks before Preserflo implantation to ensure the absence of any conjunctival hyperemia on the day of surgery. Instead, they are given oral azetacolamide for four weeks and corticoid eye drops 3 days prior to surgery. Then, 2–3 h before the operation, they receive intravenous acetazolamide and mannitol, in order to lower the pre- to postoperative pressure gradient. The subsequent surgical procedure of the implantation of the Preserflo Microshunt has been explained in detail elsewhere [9,10,15]. In short, after dissection of the conjunctiva and Tenon’s capsule, mitomycin-C (MMC) 0.2 mg/mL is applied to the bare sclera for 3 min by placing sponges into the conjunctival flap. After subsequent rinsing with a balanced salt solution, a 2 mm deep scleral tunnel is created using a 1 mm lance. A 25 gauge needle is then guided through this tract to enter the anterior chamber, thus forming a tunnel between the anterior chamber and the subconjunctival pocket 3.5–4 mm from the limbus. The microshunt is inserted ab externo into the tunnel with its tip reaching approximately 2 mm into the anterior chamber, while its wings are kept inside the scleral pocket. After confirmation of flow through the device, seen by the formation of drops at the external end of the tube, Tenon’s capsule and conjunctiva are closed. Figure 1 shows the correct postoperative placement of the Preserflo MicroShunt.

In our clinic, we regularly give 5-fluoruracil injections into the subtenonal space postoperatively if corkscrew vessels are present, or if the scleral pocket appears encapsulated. 

### 2.3. Data Collection

Data on age, gender, ethnicity, type of glaucoma, and previous ocular surgeries including laser treatments were compiled from the electronic patient files. Surgeries were defined as all interventions used to treat conditions of the eyes, including laser treatment, such as selective laser-trabeculoplasty and other MIGS procedures, as well as cataract surgery and other penetrating procedures. Clinical information included results of slit-lamp examination, best corrected visual acuity, applanatory IOP, perimetric testing results including mean deviation, lens status, number of postoperative 5-fluoruracil injections, and number of antiglaucoma medications (topical and oral). Information was assessed preoperatively and at each of five postoperative timepoints: day 1, month 1, month 3, month 8, and month 12. After discharge, patients were scheduled to revisit 1, 3, 8, and 12 months after surgery. If patients reconsulted with our clinic more often than they were scheduled to, the IOP values closest to the whole month mark were drawn into the statistical analysis of IOP development. However, unscheduled visits were considered in the calculation of success rates. The occurrence of postoperative adverse events, as well as the necessity for revision surgeries, were noted. Adverse events were defined as hypotony ≤5 mmHg at any of the given timepoints, postoperative hyphemia, choroidal detachments, vitreous hemorrhage, or opening of Tenon’s capsule. Revision surgeries were defined as surgical procedures that succeeded Preserflo implantation and that were conducted in order to achieve therapeutic success. Trabeculectomy, bleb revision, pars-plana vitrectomy, cyclophotocoagulation, implantation of another microshunt, and rinsing of the anterior chamber were regarded as revision procedures. The number of subconjunctival 5-fluoruracil injections was noted, but not counted as a revision procedure. 

Visual field testing was conducted using the automated Humphrey Visual Field Analyzer II (HFA II, model 750; Carl Zeiss Meditec AG, Jena, Germany) with the standard program of the 30–2 Swedish interactive threshold algorithm (SITA fast). 

### 2.4. Outcome Measures

The main outcome after 12 months was the overall success rate. Clinical outcome was classified as complete success (CS), qualified success (QS), or failure in accordance with the Primary Tube versus Trabeculectomy Study [16]. CS was achieved if, from month 1 onward, a patient’s IOP reached values of 6–21 mmHg on two consecutive follow-up visits with a reduction of ≥20% in comparison to mean preoperative IOP in both visits. If patients fulfilled the abovementioned criteria, but required further supplemental medical therapy, they were considered as having achieved QS. Overall success rate was defined as all cases of CS and QS. Failure was defined as IOP >21 mmHg in any of two consecutive postoperative visits after 1 month postoperatively, an IOP reduction of less than 20% on any of two consecutive postoperative visits in comparison to baseline 1 month postoperatively, a necessity for revision surgery, or loss of light perception following microshunt implantation from day 1 postoperatively. 

Additionally, overall IOP reduction (mmHg) at 12 months after surgery was investigated in comparison to preoperative values in the entire patient cohort and in the disease severity subgroups. The median IOP of the date closest to the predefined intervals was calculated and drawn into the study. Eyes were allocated to disease severity groups (early, moderate, or severe) on the basis of the results of perimetric testing (Hodapp–Parrish–Anderson classification) [17]. We further report the number of postoperative supplemental antiglaucoma medications, number of postoperative adverse events, number of postoperative 5-FU injections, and number of revision procedures within 1 year of follow-up.

### 2.5. Statistical Analysis

Statistical analyses were performed using IBM SPSS Statistics for Windows, Version 28.0 (IBM Corp.: Armonk, NY, USA). All *p*-values and confidence limits were two-sided and intended to be exploratory rather than confirmatory. Therefore, no adjustment for multiplicity was made. Exploratory two-sided *p*-values ≤ 0.05 were considered statistically noticeable.

In descriptive analysis, continuous variables are reported as the median (25% quantile–75% quantile, interquartile range (IQR)). Categorical variables are presented as absolute and relative frequencies. Subgroup comparisons for continuous variables were performed using the Kruskal–Wallis test and Fisher’s exact test for categorical variables. A comparison of pairwise IOP changes between two timepoints was performed using Wilcoxon signed-rank tests. Boxplots were used for graphical representation. Missing values were regarded as missing completely at random.

## 3. Results

### 3.1. Baseline Characteristics

A total of 160 eyes of 160 patients from the Department of Ophthalmology, University of Münster Medical Center, Germany, were included in this study. Patient characteristics are summarized in Table 1. The median follow-up time was 9 (IQR 5–12) months.

### 3.2. Outcome: Success Rates 

The overall success rate for the entire study population was 61.9% (CS: 51.3%, QS: 10.6%). Overall success was highest in patients with early glaucoma, followed by moderate and severe glaucoma. Overall success rate was lowest for secondary glaucoma patients (Table 2). The overall success rate differed noticeably among disease severity groups (*p* = 0.04) and among glaucoma subtypes (*p* = 0.02); however, it did not differ noticeably among eyes grouped according to the number of prior surgical interventions (*p* = 0.35). 

### 3.3. Outcome: IOP Reduction 

**Figure 2:**
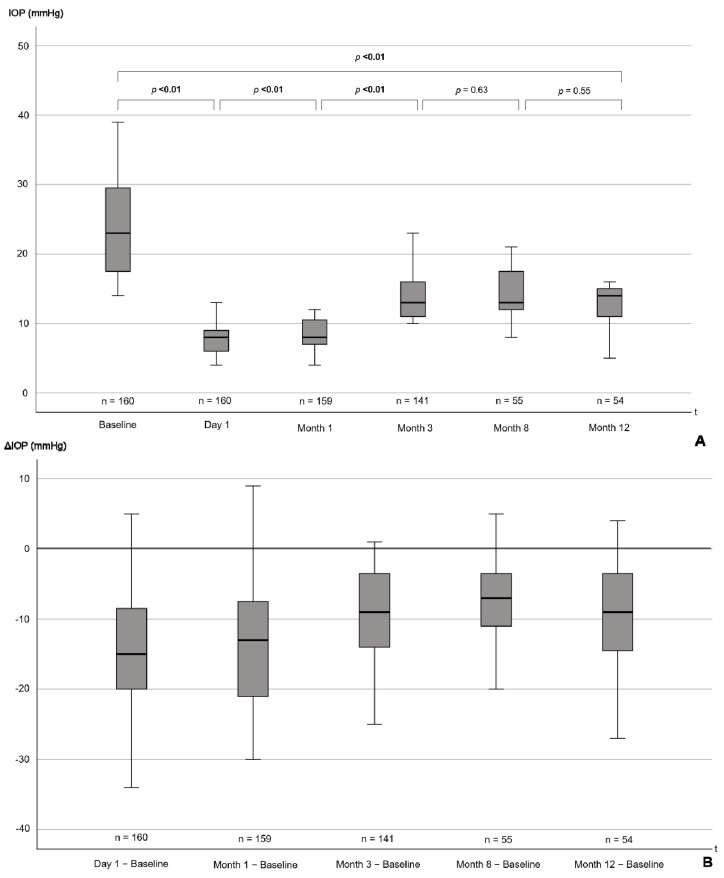
(**A**) Boxplots showing IOP from baseline to 12 months postoperatively. *p*-values for the difference between individual follow-up timepoints, as well as for the baseline comparison to 12 months, are presented. (**B**) Boxplots showing the reduction in IOP from baseline at the distinct follow-up timepoints. *p*-values are from Wilcoxon signed-rank tests. *p*-values ≤ 0.05 are highlighted in bold. Note that distances between the time intervals are not to scale. IOP = intraocular pressure, mmHg = millimeters of mercury, t = time, Δ = difference.

The median IOP reduction for the entire patient cohort over 1 year was 6 (IQR 2–13) mmHg with median IOP values of 22 (IQR 17–27) mmHg preoperatively and values of 14 (IQR 12–16) mmHg at 12 months postoperatively (*p* < 0.001; Figure 2, Table 3). 

**Figure 3:**
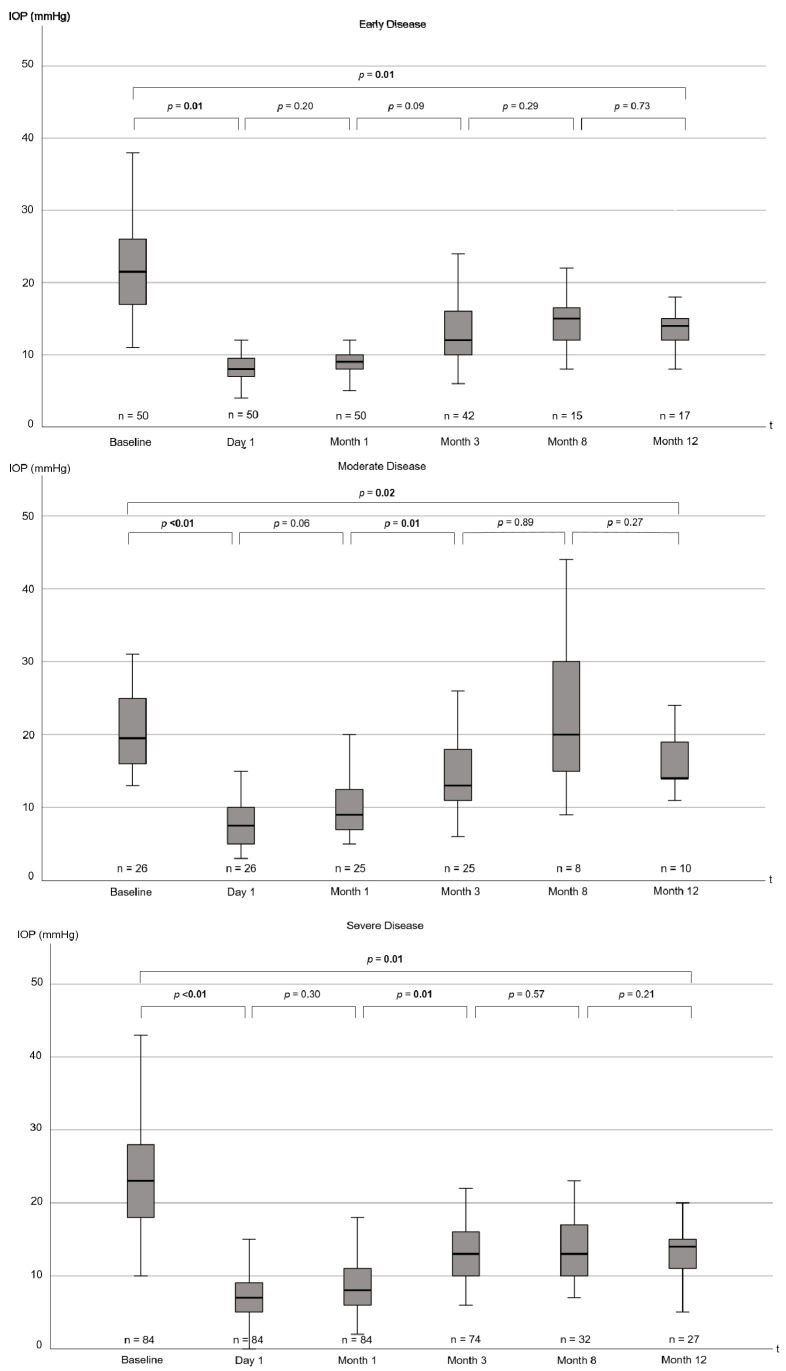
Boxplots showing IOP from baseline to 12 months postoperatively for disease severity cohorts (early, moderate, and severe). Note that distances between the time intervals are not to scale. *p*-values for the difference between individual follow-up timepoints, as well as for the comparison of baseline to the 12 month follow-up date, are displayed. *p*-values are from Wilcoxon signed-rank tests. *p*-values ≤ 0.05 are highlighted in bold. IOP = intraocular pressure, mmHg = millimeters of mercury, t = time.

IOP was noticeably reduced at 12 months postoperatively irrespective of disease severity group or type of glaucoma (Table 3, Figure 3). The median IOP reduction differed noticeably among disease severity groups (*p* < 0.01) and glaucoma subtypes (*p* = 0.01).

### 3.4. Postoperative Development

#### 3.4.1. Supplemental Medications

Changes in supplemental medications were analyzed for the 54 patients who were present at the 12 month follow-up visit. The number of supplemental medications decreased noticeably from three (IQR 2.8–4) medications at baseline to zero (IQR 0–2) medications at 12 months postoperatively (*p* < 0.01).

#### 3.4.2. Postoperative Complications

Postoperatively, 87 eyes (54%) were affected with hypotony (IOP ≤ 5 mmHg), with 30 eyes (19%) showing a choroidal detachment. Central choroidal detachment was observed in three eyes (2%). In the majority of cases, hypotony and choroidal detachment resolved spontaneously or with support of intensified locally applied steroidal therapy in the first weeks postoperatively. Hypotony persisted after 1 week in 18 eyes (11%) and after 90 days in four eyes (2%). Hyphemia was observed in 38 eyes (24%) (Table 4). 

The number of eye-related adverse events differed noticeably among disease severity groups (*p* = 0.05) and glaucoma subtypes (*p* = 0.01), but did not differ noticeably among eyes grouped according to the number of prior surgical interventions (*p* = 0.72) (Table 4). 

We would like to highlight two cases with unusual problems following Preserflo implantation that happened in our tertiary referral center. 

One patient suffered from bleb infection with conjunctival necrosis followed by Preserflo explantation and scleral patch. The patient’s history contained herpetic keratouveitis, and XEN Implant in the same eye. The conjunctiva was avascular prior to the Preserflo implantation, which likely predisposed to infection and necrosis.

Another patient with secondary glaucoma after perforating eye injury, aphakia, and pretreatment with two cyclophotocoagulations developed hypotony and phthisis bulbi in the further course after Preserflo implantation. The patient required numerous further procedures, including perforating keratoplasty, Eckhardt prosthesis, and vitrectomy. As a result, in our opinion, phthisis bulbi should be regarded as a result of the perforating damage rather than a complication of Preserflo implantation.

#### 3.4.3. 5-FU Injections and Postoperative Interventions

A total of 123 eyes (77%) received at least one subconjunctival injection of 5-FU (range: 0–21; median: 2) (Table 5). We did not count subconjunctival injections of 5-FU as revision surgery. Furthermore, 73% of all 5-FU injections were given within 1 week after surgery, 88% were given within 14 days after surgery, 97% were given within the first month after surgery, and 100% were given within the first 3 months after surgery. 

Overall, 40 patients (25%) needed ophthalmological revision surgery. Bleb revision surgery was required in 28 eyes (18%). A total of 12 eyes (8%) underwent anterior chamber surgery, due to hyphemia or hyperfiltration with shallow anterior chamber. Subsequent trabeculectomy was performed in five eyes (3%), and cyclophotocoagulation was performed in six eyes (4%). Postoperative vitrectomy was performed in three eyes, due to vitreous body prolapse (2%) (Table 5). The percentage of revision surgeries differed noticeably among disease severity groups (*p* = 0.01), glaucoma groups (*p* < 0.01), and eyes with differences in the number of previous surgical interventions (*p* = 0.01) (Table 5). 

## 4. Discussion

In this retrospective study, the Preserflo MicroShunt achieved an overall success rate of 61.9% (CS: 51.3%, QS: 10.6%) in a heterogenous glaucoma cohort. Median IOP reduction was 6 (IQR 2–13) mmHg after 12 months, and revision surgery was required in 25% of all cases. Postoperative adverse events were noted in almost two-thirds of patients. Compared to baseline, the number of supplemental glaucoma medications taken 12 months postoperatively was noticeably lower in the 54 patients who were present at 12 months follow-up.

Among the first trials to report success rates for this novel concept, Battle et al. achieved an overall success rate of 100% after 1 year of follow-up, which has since not been replicable [18]. Consisting of only 23 patients, the overall success rate of their Preserflo trial remains among the highest in the literature, with consistent overall success rates ≥ 91% throughout a 3 year follow-up period [8]. Meanwhile, the CS rate was high with 91% after 1 year.

Success rates of later trials with bigger study cohorts ranged 53.9% to 92.3% for CS and 68.3% to 92.6% for overall success rates [7,9,10,11,12,13]. These numbers are similar to success rates in this trial. Some of these authors further distinguished among different types of glaucoma. Nobl et al. reported success rates for both PEX glaucoma patients and POAG glaucoma patients. Interestingly, both CS and QS were comparable between PEX patients and POAG patients, which contrasts earlier studies reporting on success rates in penetrating glaucoma surgery, such as trabeculectomy [19]. The authors attribute this observation to the fact that the minimally invasive approach does not aggravate the already compromised blood–aqueous barrier in PEX glaucoma as much as penetrating glaucoma surgery, resulting in lower inflammatory cytokine levels in the anterior chamber and, as a result, lower rates of fibrosis and scarring [7,20].

Conversely, in this trial, patients with POAG had higher overall success rates than PEX glaucoma patients. PEX patients had a noticeably higher proportion of QS and failure cases than the POAG cohort. It should, however, be noted that comparability among subpopulations in this trial is limited, as patient characteristics across subgroups were not matched; thus, parameters, such as age, gender, or disease severity might explain the differences seen here. Nevertheless, this explorative analysis allows an estimate of real-world performance of the Preserflo Microshunt system in different glaucoma group constellations. We observed secondary glaucoma patients to have the lowest overall success rate, despite featuring the highest median IOP decrease. This is because the rate of revision surgery in secondary glaucoma eyes was the highest of all with 44%. We assume that, as glaucoma in these patients developed secondary to another underlying cause, the surgical procedure oftentimes is more complicated, and the postoperative development is influenced by factors not present in other types of glaucoma. Success rates for pigment-dispersion and primary angle-closure glaucoma eyes were high, yet the small population in these cohorts limits the generalizability of this observation. 

The level of IOP reduction after 1 year in this trial is comparable to other trials [10,11,21,22]. When differentiating among disease severity groups, IOP reduction at 12 months follow-up was greatest in patients with severe glaucoma. This might in part be explained by the behavior of ophthalmologists during postoperative follow-up visits, as most experts might tend to prescribe supplemental, IOP-lowering medication in patients with advanced optical nerve damage rather than in patients with an early form of glaucoma.

Schlenker et al. reported secondary OAG and a lower dose of intraoperative MMC to be associated with a higher failure rate [9]. This association might in part explain the comparatively low success rate reported by Baker et al., who used a low dosage of MMC in their trial (0.2 mg/mL) [12]. Durr et al. investigated the influence of different concentrations of MMC and found that a lower dosage of MMC was associated with higher rates for needling [11]. They also found that the association between disease severity and failure rate was high in mild to moderate disease [11]. This is in line with reports by Tanner et al., who described an association between higher mean deviation and failure [10]. However, in this trial, this association was not as present, as described by previous studies. Although CS rates were higher in less advanced glaucoma stages, failure rates were comparable among all severity groups. This deviation from previous reports might be attributable to the composition of the population in this study, as it was more heterogeneous than in other trials, which oftentimes only included one type of glaucoma.

The rate of postoperative hypotony in this trial (54%) was high in comparison to other studies, with highest rates not exceeding 40% [7,8,9,11,12,18,23]. Likewise, choroidal detachment also occurred noticeably more often in this trial (19%), compared to most reports in the literature [8,9,11,18,23], with only Nobl et al. reporting higher choroidal detachment rates of 30% for PEX glaucoma patients [7]. Central choroidal detachment occurred in three cases (2%) in this trial, which is comparable to rates reported in other studies [7,13]. We report real-world data of patients treated at a tertiary care referral center. Therefore, the number of complicated glaucoma cases, e.g., involving eyes with secondary glaucoma or with history of previous glaucoma surgery, was naturally high and might at least in part explain the large number of cases with postoperative hypotony. The frequent usage of 5-FU in this study might also have contributed to this observation. Likewise, the pre-operative administration of acetazolamide and mannitol might have had an influence on postoperative IOP development. With a half-life of 6–8 h [24], intravenous acetazolamide might have affected the IOP in our study population within the first days after surgery. Nonetheless, over the course of 1 week, hypotony resolved in 89% of cases and in 95% of affected cases after 3 months without any sequelae.

The rates for revision surgery were higher in our study than reported by Nobl et al. [7] and Durr et al. [11], albeit lower than that described by Baker et al., who reported a rate of 40.8% of patients requiring postoperative interventions [12]. Comparison of postoperative revision procedures is difficult across studies, as there is a wide variety of what authors consider a postoperative intervention. Tanner et al. separately reported the rate of bleb revisions to be 11.5% in their study [10], which is lower than in our study (17.5%). We included a proportionally large number of patients who had undergone previous ocular surgery (76%), in comparison to the 66.3% of patients in the trial by Tanner et al. [10]. The difference in baseline study characteristics might explain the differences in postoperative bleb revisions seen in this particular case and in other postoperative procedures. Eventually, the decision to perform surgical revision is not standardizable and is left to the discretion of the treating surgeon, making comparison with other trials, even with those that apply the same definitions, difficult.

### Limitations

This study had some limitations. Firstly, due to its retrospective design, we are limited in the possibility to comment on future IOP development. Although we do not expect IOP and, therefore, success rates to fluctuate strongly in the period after 12 months of follow-up, recent trials have seen a decrease in success rates over a follow-up period of 5 years [8]. Further longitudinal studies with larger follow-up periods are required for adequate prognoses. 

Secondly, even though we accounted for most of the factors known to exert an influence on glaucoma surgery results, such as age, sex, severity of glaucoma, type of glaucoma, and previous surgery, individual factors and individual postoperative behaviour might have had an influence on IOP development. Future studies are needed to replicate the findings reported in this trial. 

Thirdly, we saw a relatively large dropout rate after the third postoperative month. In our experience, this very well represents the clinical reality in postoperative care. Patients with insufficient IOP regulation after surgery will usually attend most, if not all, prescheduled follow-up visits. However, those patients with an unproblematic postoperative development tend to not keep further appointments from a certain point onward if they do not see the necessity to revisit. The overall patient compliance should, therefore, be kept in mind when interpreting the results depicted in this study. 

## 5. Conclusions

To summarize, the Preserflo MicroShunt achieved an overall success rate of 61.9% in a study cohort consisting of patients with various types and severity stages of glaucoma. It showed noticeable reductions in both IOP and number of medications. The Preserflo device is still new to many professionals; therefore, comparison to other established invasive glaucoma treatments is limited. As surgeons become more experienced in the implantation of the system, long-term outcomes might change.

## Figures and Tables

**Table 1:** General patient characteristics. Data are presented as the median (25% quartile–75% quartile) or as absolute and relative values.

Characteristics	Total Cohort
Eyes (*n*)	160
Patients (*n*)	160
Age (years)—median (IQR)	69 (62–77)
Gender (M:F) (*n*,%)	80 (50%):80 (50%)
Study eye (R:L) (*n*; %)	83 (52%):77 (48%)
Disease severity groups (*n*; %)	
Early	50 (31%)
Moderate	26 (16%)
Severe	84 (53%)
Type of glaucoma (*n*, %)	
POAG	111 (69%)
PEX glaucoma	28 (18%)
Secondary glaucoma	9 (6%)
Pigment-dispersion glaucoma	8 (5%)
Primary angle-closure glaucoma	4 (2%)
Previous surgery (*n*, %)	
None	38 (24%)
Total	122 (76%)
1–2 operations	85 (53%)
>2 operations	37 (23%)

*n* = number, % = percentage, M = male; F = female, R = right, L = left, POAG = primary open-angle glaucoma, PEX = pseudo-exfoliation.

**Table 2:** Success and failure rates for the entire study population and according to disease severity groups, type of glaucoma, and number of previous surgeries. Data are presented as absolute and relative values.

	Complete Success	Qualified Success	Failure
Total study population, (*n*, %)	82 (51%)	17 (11%)	61 (38%)
Disease severity groups			
early (*n*, %)	29 (58%)	4 (8%)	17 (34%)
moderate (*n*, %)	14 (54%)	1 (4%)	11 (42%)
severe (*n*, %)	39 (46%)	12 (14%)	33 (39%)
Type of glaucoma			
POAG (*n*, %)	65 (59%)	9 (8%)	37 (33%)
PEX glaucoma (*n*, %)	9 (31%)	5 (18%)	14 (50%)
Secondary glaucoma (*n*, %)	2 (22%)	0 (0%)	7 (78%)
Pigment-dispersion glaucoma (*n*, %)	3 (38%)	3 (38%)	2 (25%)
Primary angle-closure glaucoma (*n*, %)	3 (75%)	0 (0%)	1 (25%)
Previous surgical interventions			
None	21 (53%)	5 (13%)	14 (35%)
1–2	24 (49%)	7 (14%)	18 (37%)
>2	37 (52%)	5 (7%)	29 (41%)

*n* = number, % = percentage, POAG = primary open-angle glaucoma, PEX = pseudo-exfoliation.

**Table 3:** IOP reduction at 12 months postoperatively in comparison to baseline for the entire patient population and according to disease severity groups, type of glaucoma, and number of previous surgeries. Data are presented as numbers and medians (25–75% quantile). *p*-values are reported from the Wilcoxon signed-rank test. *p*-values < 0.05 are highlighted in bold.

	IOP Baseline	IOP 12 Months	IOP Reduction	*p*-Value
Total study population (mmHg)	*n* = 160 22 (17–27)	*n* = 54 14 (12–16)	*n* = 54 6 (2–13)	**<0.01**
Disease severity groups				
Early (mmHg)	*n* = 50 21 (17–26)	*n* = 17 14 (12–15)	*n* = 17 4 (1–10)	**0.01**
Moderate (mmHg)	*n* = 26 19 (16–27)	*n* = 10 14 (14–19)	*n* = 10 4 (2–5)	**0.02**
Severe (mmHg)	*n* = 84 23 (18–28)	*n* = 27 14 (11–15)	*n* = 27 10 (2–14)	**0.01**
Type of glaucoma				
POAG (mmHg)	*n* = 111 21 (17–27)	*n* = 37 14 (12–16)	*n* = 37 6 (1–11.3)	**<0.01**
PEX (mmHg)	*n* = 28 24 (22–28)	*n* = 9 13 (13–14)	*n* = 9 9 (4–13.5)	**<0.01**
Secondary (mmHg) *	*n* = 9 26 (24–29)	*n* = 4 20 (15–24)	*n* = 4 11 (7.3–14.3)	
Pigment dispersion (mmHg) *	*n* = 8 18 (17–22)	*n* = 2 14 (14–14)	*n* = 2 3 (3–5)	
Primary angle closure (mmHg) *	*n* = 4 22 (18–28)	*n* = 2 8 (8–8)	*n* = 2 8 (3–15.8)	
Previous surgical interventions				
None	*n* = 40 23 (20–26)	*n* = 10 14 (10–16)	*n* = 10 10 (7–11)	**<0.01**
1–2	*n* = 49 23 (17–28)	*n* = 13 15 (13–17)	*n* = 13 13 (6–15)	**<0.01**
>2	*n* = 71 21 (17–28)	*n* = 21 14 (12–15)	*n* = 21 3 (1–6)	**0.01**

mmHg = millimeter mercury, POAG = primary open-angle glaucoma, PEX = pseudo-exfoliation. * *p* value not calculated, due to the small sample size.

**Table 4:** Number of eye-related postoperative adverse events in the entire study population and according to disease severity groups, type of glaucoma, and number of previous surgeries within 1 year. Data are presented as absolute and relative values.

Eye-Related Postoperative Adverse Events	*n* (%)
Total	106 (66%)
Hypotony (IOP ≤ 5 mmHg)	87 (54%)
Hypotony (IOP ≤ 5 mmHg) persistent after 1 week	18 (11%)
Hypotony (IOP ≤ 5 mmHg) persistent after 3 months	4 (3%)
Choroidal detachment	30 (19%)
Central choroidal detachment	3 (2%)
Peripheral choroidal detachment	27 (17%)
Hyphemia	38 (24%)
Disease severity groups	
Early (*n* = 50)	34 (68%)
Moderate (*n* = 26)	15 (58%)
Severe (*n* = 84)	57 (68%)
Type of glaucoma	
POAG (*n* = 111)	71 (64%)
PEX (*n* = 28)	20 (71%)
Secondary (*n* = 9)	7 (78%)
Pigment dispersion (*n* = 8)	6 (75%)
Primary angle closure (*n* = 4)	2 (50%)
Previous surgical interventions	
None (*n* = 40)	26 (65%)
1–2 (*n* = 49)	32 (65%)
>2 (*n* = 71)	48 (68%)

*n* = number, % = percentage, POAG = primary open-angle glaucoma, PEX = pseudo-exfoliation.

**Table 5:** Number of postoperative 5-FU injections and revisory operations in the entire study population within 1 year. Results are reported as numbers and percentages.

Postoperative Interventions within 1 Year	*n* (%)
Subconjunctival injection of 5-FU, total	123 (77%)
None	37 (23%)
1–3	83 (52%)
≥4	40 (25%)
**Revision surgeries within 1 year**	***n* (%)**
Total	40 (25%)
Anterior chamber surgery	12 (8%) *
Bleb revision surgery	28 (18%) *
Trabeculectomy	5 (3%) *
Cyclophotocoagulation	6 (4%) *
Vitrectomy	3 (2%) *
Percentage of revision surgeries by disease severity group	
Early (*n* = 50)	9 (18%)
Moderate (*n* = 26)	8 (31%)
Severe (*n* = 84)	23 (27%)
Percentage of revision surgeries by type of glaucoma	
POAG (*n* = 111)	25 (23%)
PEX glaucoma (*n* = 28)	11 (39%)
Secondary glaucoma (*n* = 9)	4 (44%)
Pigment-dispersion glaucoma (*n* = 8)	0
Primary angle-closure glaucoma (*n* = 4)	0
Percentage of revision surgeries by number of previous surgical interventions	
None (*n* = 40)	8 (20%)
1–2 (*n* = 49)	16 (33%)
>2 (*n* = 71)	16 (23%)

*n* = number, % = percentage, POAG = primary open-angle glaucoma, PEX = pseudo-exfoliation. * Percentage values reported as the proportion of the entire study population (*n* = 160).

## Data Availability

Not applicable.
